# Study on Preparation and Performance of Aerated Concrete Using Spodumene Mining Residue as Silicious Material

**DOI:** 10.3390/ma18050957

**Published:** 2025-02-21

**Authors:** Xiaoying Li, Qiang Zeng, Zhongtao Zhu, Jie Ren, Zhongyuan Lu

**Affiliations:** 1 State Key Laboratory of Environment-Friendly Energy Materials, School of Materials and Chemistry, Southwest University of Science and Technology, Mianyang 621010, China; 2 The Second Branch of Chongqing-Wan High-Speed Railway, China Railway 18th Bureau Group Corporation Limited, Wanzhou District, Chongqing 404100, China; 3 China Nuclear Industry Huaxing Construction Co., Ltd., Nanjing 210019, China

**Keywords:** spodumene mining residue, aerated concrete, mechanical property, pore structure, interaction mechanism

## Abstract

In this research, the spodumene mining residue was used as siliceous material, completely replacing quartz sand, to prepare aerated concrete. The mechanical properties, pore structure, hydration characteristics of the aerated concrete, and the spodumene mining residue–cement paste interaction mechanism were studied by orthogonal experiment, X-ray diffraction, Fourier-Transform Infrared Spectroscopy, thermogravimetry, and mercury-injection test methods. The result showed that the water–cement ratio significantly affected the mechanical properties and dry density of the aerated concrete. The content of aluminum powder paste, spodumene mining residue, and water-cement ratio significantly affected the pore structure of aerated concrete. The pore size was mainly distributed in the range of less than 100 nm in hardened samples. The main hydration products of the aerated concrete containing spodumene mining residue were xonotlite, tobermolite, and C-S-H gel (or its derivatives). Spodumene mining residue had a small amount of active silicon and aluminum components, which could be motivated by an alkaline environment. In the simulation pore solution, the weak pozzolanic reaction was produced to generate C-S-H and its derivatives, which adhered to the surface of the spodumene mining-residue particle and filled in the interface between spodumene mining residue and cement paste, to improve the density of aerated concrete.

## 1. Introduction

In recent years, the development of Chinese power batteries and the new-energy vehicle industry has greatly promoted the consumption demand of raw materials such as lithium carbonate and lithium hydroxide [1,2]. According to statistics, in 2021, the global ore production capacity of lithium hydroxide was about 350,000 tons. The production capacity of lithium hydroxide in Sichuan and Jiangxi regions in China was about 110,000 tons and 133,000 tons, respectively, accounting for 32 wt% and 38 wt% of the global total production capacity. This will undoubtedly increase the amount of lithium mining [3,4,5]. The types of lithium resources in China mainly include the lithium mine in salt-lake, spodumene and lepidolite ore. The lithium resources in the Sichuan region account for about 40 wt% of the total lithium resources in China, which are mainly spodumene ore. However, more mining residues would be generated during the mining process of spodumene and directly deposited in waste rock storage, resulting in a waste of land resources, and more dust, endangering the health of surrounding residents. Therefore, it is urgent to explore the recycling ways of spodumene mining residue.

It is worth noting that spodumene mining residue is different from lithium slag, which is a byproduct of the process of producing lithium carbonate by sulfuric acid after high-temperature roasting of spodumene ore [6]. At present, the research on the resource utilization of spodumene mining residue includes lithium resource extraction by heavy medium separation [7], use as filling material [8], and resource utilization as building materials [9,10,11]. Among them, the resource utilization of building materials can effectively improve the utilization rate of spodumene mining residue, but the preparation of glass and ceramics will bring significant energy consumption and environmental load.

The spodumene mineral phase is often associated with quartz, mica, pyroxene or feldspar. Spodumene mining residue has a high content of silicon and aluminum, of which silicon content usually exceeds 65% (measured by SiO_2_), and is expected to be used as a silicic raw material in cement-based materials. Spodumene mining residue has a high crushing value and poor hardness, and is not suitable for use as aggregate in high-strength-grade cement-based materials. With the improvement in building energy saving and functional requirements, the attention to porous cement-based materials such as aerated concrete and foamed concrete is increasing. Spodumene mining residue is expected to replace quartz sand used in aerated concrete. The use of spodumene mining residue in cement-based materials could effectively inhibit the alkali–silica reaction, and it was also proved that spodumene mining-residue aggregate in cement-based materials could improve the interface structure of aggregate and cement paste [12]. However, the preparation and properties of cement-based materials containing spodumene mining residue need to be further studied. In particular, the interaction mechanism between spodumene mining residue and cement paste remains unclear.

Therefore, the spodumene mining residue was used as siliceous raw material to prepare aerated concrete, in this paper. The mechanical properties, pore structure and hydration characteristics of the aerated concrete will be studied. The interaction mechanism between spodumene mining residue and cement paste will be analyzed by XRD, TG, FTIR and simulated pore-solution soaking method. This work is expected to explore a new way of the resource utilization of spodumene mining residue, and also to provide theoretical support for spodumene mining-residue siliceous-material use in aerated concrete. In addition, this research is expected to reveal the mechanism of spodumene mining residue aggregate and cement paste.

## 2. Raw Materials and Experimental Program

### 2.1. Raw Materials

**Figure 1:**
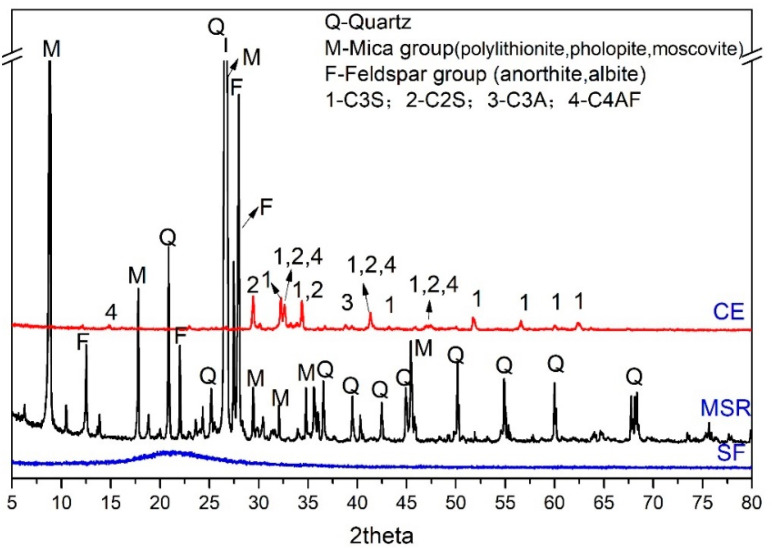
The mineral composition of raw materials.

In this research, the P.O42.5 cement (CE, commercial, Lafarge Cement, Mianyang, China), spodumene mining residue (SMR, typical spodumene mining area of Sichuan, Sichuan, China), lime (LI, commercial, Chuanash Biotechnology Co., LTD, Yibin, China), gypsum (GY, commercial, Sichuan Lishen Building Materials Group Co., LTD, Deyang, China), silica fume (SF, commercial, Yixiang new material Co., LTD, Henan, China), and aluminum powder paste (AP, commercial, Chengdu construction construction Cely concrete Co., LTD, Chengdu, China) were used as raw materials. Sodium hydroxide, potassium hydroxide, calcium hydroxide, sodium sulfate and sodium silicate were used as chemical reagents. The chemical composition and mineral composition of raw materials are shown in Table 1 and Figure 1. The purity of chemical reagents was listed in Table 2. From Figure 1, the mineral composition of spodumene mining leftover is quartz, the mica group and the feldspar group, the mineral composition of cement is C_3_S, C_2_S, C_3_A and C_4_AF, and the mineral composition of silica fume is amorphous.

**Figure 2:**
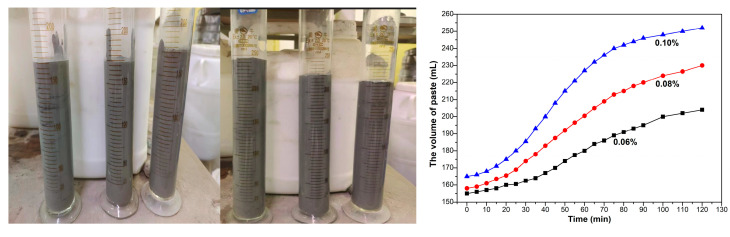
Test process diagrams, and paste volume under different aluminum contents and curing times.

Figure 2 showed the gas production process of aluminum paste. With the extension of reaction time, the production volume of gas of aluminum powder paste increased quickly at first, then increased slowly, then entered a stable state after reaction for 120min. A total of 1g aluminum paste would produce about 8.5 mL gas in cement paste.

### 2.2. Mixing Proportion and Preparation

Table 3 lists the mixing proportion and curing condition of aerated concrete containing spodumene mining residue. The SF-SMR ratio (the content ratio of silica fume and spodumene mining residue), aluminum powder pastes and water–cement ratio were set as variables for the orthogonal design of the experiment. And the sum content of SF and SMR was fixed as 68 wt% of the total content. The volume of aerated concrete samples was 70.7 mm × 70.7 mm × 70.7 mm. The preparation, curing and properties testing of aerated concrete were all according to the standard GB/T 11969-2020 (in Chinese) [13].

### 2.3. Characterization

**Figure 3:**
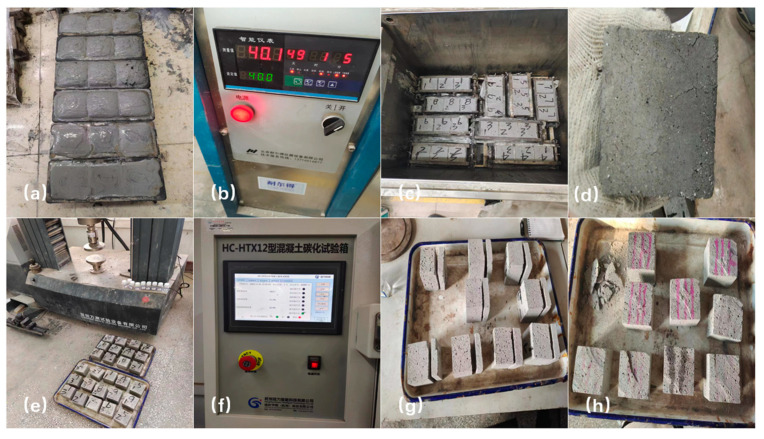
The flow chart of aerated concrete preparation and performance test. (**a**) the block after casting (**b**) curing condition and equipment (**c**) curing process (**d**) the sample after curing (**e**) test the strength of samples (**f**) test conditions and equipment for carbonization resistance experiment (**g**) samples treatment before carbonization resistance test (**h**) morphology of samples after carbonization resistance test.

The compressive strength of aerated concrete was tested using the TSH10A instrument (Shenzhen Wance Testing Machine CO., Ltd., Shenzhen, China) with a loading rate of 2.0 kN/s ± 0.5 kN/s. Carbonation resistance property of aerated concrete was tested according to the standard GB/T 11969-2020 (in Chinese). After mechanical properties testing, the sample blocks were collected, dried, crushed or ground using XRD, SEM, MIP, FTIR, and TG analysis. The chemical composition of raw materials was tested by X-ray fluorescence analysis (XRF, Axios, PANalyticalB.V., Almelo, The Netherlands) with the maximum power, accuracy and content range of 2.4 kW, 0.0025° and 0.01% to 100%, respectively. The mineral composition of samples was tested by X-ray diffraction analysis (XRD, DMAX1400, Rigaku corporation, Tokyo, Japan) with the scanning range, scanning speed and step size of 3–80° (2 theta), 8°/min and 0.02°, respectively. XRD patterns were analyzed according to the inorganic crystal structure database (ICSD) and crystallography database. Scanning electron microscopy (SEM, Ultra55, Carl Zeiss NTS GmbH, Oberkochen, Germany) was used to investigate the morphology of aerated concrete samples. Mercury intrusion porosimetry (MIP, Autopore IV9500, Micromeritics Co., Ltd., Norcross, GA, USA) was used to study the pore size distribution of aerated concrete samples. Thermogravimetric analysis (TGA) was performed to predict the weight loss of hydrated aerated-concrete samples by the analyzer (STA449F5, Germany Netzsch Instrument Manufacturing Co., Ltd., Selb, Germany). The dry samples (12 ± 3 mg) were tested at the temperature range from 50 °C to 1000 °C, a 20 °C/min heating rate and in a nitrogen measurement atmosphere. To identify the weight loss of aerated concrete samples, the first derivative of the thermogravimetric (DTG) curve was used. At the same time, Fourier-transform infrared spectroscopy (FTIR, spectrum one, PerkinElmer Co., Ltd., Waltham, MA, USA) was used to identify the molecular structure of aerated concrete. The aerated concrete preparation, sample collection and the process of mechanical and carbonation resistance properties are shown in Figure 3.

## 3. Result and Discussion

### 3.1. Dry Density and Mechanical Properties of Aerated Concrete Containing Spodumene Mining Residue

**Figure 4:**
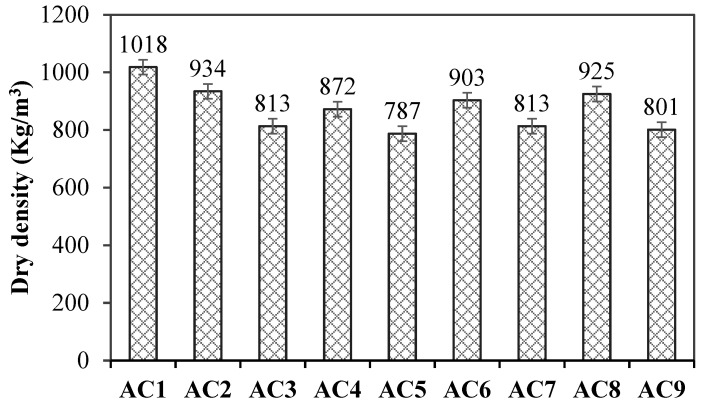
The dry density of aerated concrete containing spodumene mining residue.

Figure 4 shows the dry density of aerated concrete containing spodumene mining residue. The results of orthogonal experiments were analyzed using statistical product and service solutions (SPSS, Chinese version 11.0, Updated at August 2024) software. The ranking of influencing factors of concrete density was the water–solid ratio, aluminum powder paste content and SF-SMR ratio. In other words, the water–solid ratio had the greatest influence on the dry density of aerated concrete. With the increase in water–solid ratio, the dry density of aerated concrete decreased, observably. In this paper, the strength value per unit was used to characterize the mechanical properties of aerated concrete.

**Figure 5:**
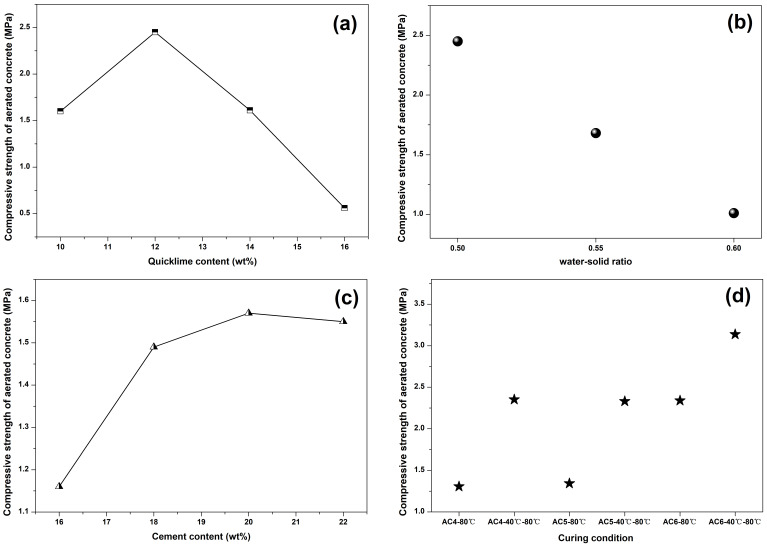
The influence of different factors on aerated concrete compressive strength. (**a**) lime content (**b**) water solid ratio (**c**) cement content (**d**) curing condition.

Figure 5 showed the influence of lime content, water–solid ratio, cement content and curing condition on the compressive strength of aerated concrete samples. In Figure 5a, c, the optimum content of lime and cement are 12 wt% and 20 wt%, respectively. Cement and lime in cement-based pastes not only provided the raw materials for hydration reaction, but also provided the reaction alkaline environment for other substances in a system such as supplementary cementitious material and aluminum powder paste. On one hand, the lower content of cement or lime was less conducive to hydration product generation and the compact structure of the aerated concrete matrix, because of the absence of calcareous materials. On the other hand, the low content of cement or quicklime led to low alkalinity of the system, which was not conducive to the gas production by aluminum powder paste. However, the excessively high content of cement and lime led to higher alkalinity and more heat release in the early stage, which resulted in a sharp increase in gas production and excessive cracks in samples. The optimum compressive strength of the aerated concrete samples containing spodumene mining residue exceeded 3 MPa.

In Figure 5b, with the increase in the water–solid ratio, the compressive strength of aerated concrete is significantly decreased. A large amount of water in the paste, resulting in a decrease in reaction concentration and more pores being left in the dry samples after water volatilization, which led to hydrated aerated-concrete-structure compactness and insufficient strength. In Figure 5d, we choose the AC_4_, AC_5_, AC_6_ mix proportion as the experimental group; two curing systems were set, namely, curing at 80 °C for 48 h and curing at 40 °C for 24 h, plus curing at 80 °C for 24 h, respectively. The result showed that the combined curing condition was more beneficial to the strength improvement of aerated concrete. High-temperature curing at the early stage of concrete would lead to the destruction of the matrix structure due to temperature stress. At the same time, the higher the curing temperature, the faster the water loss, which led to drying shrinkage and micro-cracks in aerated concrete.

### 3.2. Hydration Products, Pore Structure and Carbonation Resistance of Aerated Concrete Containing Spodumene Mining Residue

The properties of aerated concrete containing spodumene mining residue were closely related to the hydration characteristic and microstructure of cement-based systems.

**Figure 6:**
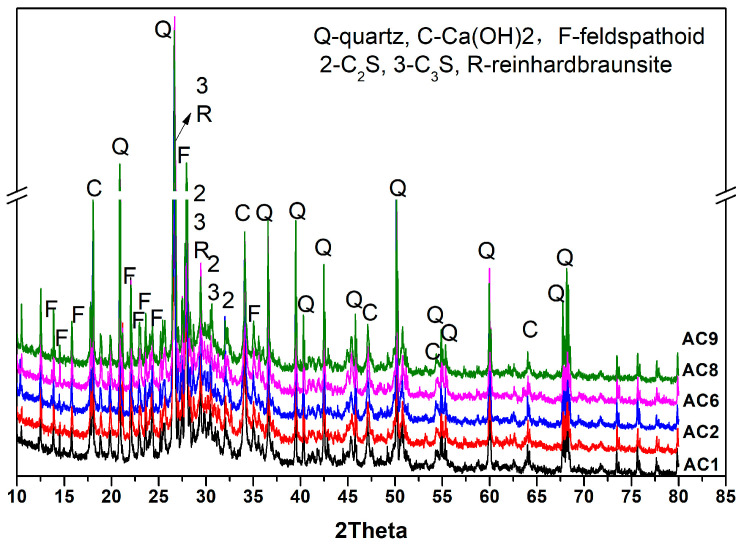
Mineral composition of hydrated aerated-concrete samples.

Figure 6 shows that the mineral composition of hydrated AC_1_, AC_2_, AC_6_, AC_8_, AC_9_ aerated concrete samples were quartz, CH, feldspathoid, reinhardbraunsite, C_3_S and C_2_S. There was no significant difference in the types of hydration products among the experimental groups. There were still unhydrated cement particles in aerated concrete samples after combined curing for 48 h (curing at 40 °C for 24 h and 80 °C for 24 h). However, the degree of crystallization of calcium hydroxide was slightly different between each experimental group, which confirmed that compositional differences still exerted an effect on the hydration of the matrix. However, due to the complex composition of the samples and poor crystallization of hydration products, it was hard to quantitatively analyze the hydration products of different experimental groups from the XRD test results.

**Figure 7:**
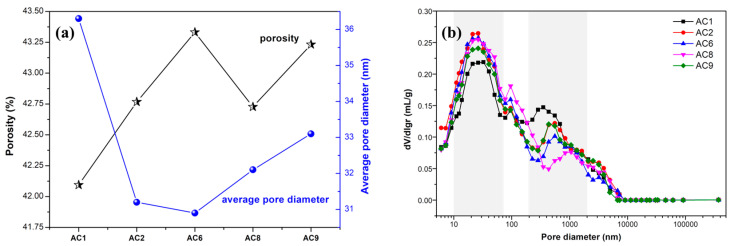
The porosity, average pore diameter and pore size distribution of aerated concrete samples. (**a**) Porosity and average pore diameter. (**b**) Pore size distribution.

The performance of aerated concrete was significantly affected by its pore structure [14,15,16]. The pore structures of the hardened samples were characterized by MIP. Figure 7a shows the porosity and average pore diameter of aerated concrete containing spodumene mining residue. The total porosity of the samples of each group ranged from 42.09% to 43.33%, and the average pore diameter of the samples of each group ranged from 30.9nm to 36.3nm. The larger the average pore diameter of cement-based porous material, the more obvious the coalescing phenomenon between bubbles in the fresh paste. Also, the porosity and mean pore diameter of cement-based materials were inversely proportional to the strength of the materials. AC1 and AC9 had the minimum and maximum porosity values, respectively, which was mainly attributed to the influence of water–solid ratio and the content of gas agent aluminum-powder paste. AC6 had the minimum average pore diameter, which was mainly attributed to the lower water–solid ratio and more silicon fume. The higher content of spodumene mining residue was unfavorable to reducing the average pore diameter of cement-based porous materials.

Figure 7b shows the pore size distribution of the AC_1_, AC_2_, AC_6_, AC_8_, AC_9_ group aerated-concrete samples. There were two obvious peaks in the pore-size-distribution curves, which showed that the pore size of the samples was mainly concentrated in two intervals of 10 nm to 100 nm and 300 nm to 1000 nm. In general, the pore size was divided into five types, namely, micropores, small pores, medium pores, large pores and cracks, which corresponded to pore-size ranges less than 10 nm, 10 nm to 100 nm, 100 nm to 1000 nm, 1000 nm to 100,000 nm and greater than 100,000 nm, respectively. It was not hard to see that most of the possible pores of the samples were distributed in the small-pore interval.

Lime and cement were the main calcium materials in the system, and their content had a significant influence on the hydration process. When appropriate contents of cement and lime were added, the hydration products of the aerated concrete such as calcium hydroxide and C-S-H gel filling pores and reducing the porosity and pore diameter, resulting in a density and strength increase. However, spodumene milling waste had a negative effect on the hydration of aerated concrete. With the increase in spodumene-milling waste powder, the hydration rate slowed down, resulting in a weakening of the capacity of hydration products to fill pores, which led to larger porosity and more large pores in hardened samples.

**Figure 8:**
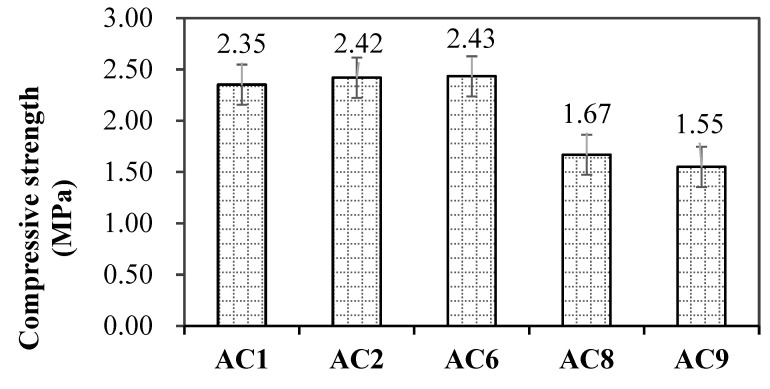
Compressive strength of aerated concrete after complex steam curing for 48 h.

Pore structure and pore size distribution significantly affected the mechanical properties of porous materials. Figure 8 showed the compressive strength of aerated concrete after complex steam curing for 48 h. AC1, AC2 and AC3 had a similar compressive strength, and AC8 and AC9 had the lower value. Combined with the pore structure characteristics of aerated concrete with SMR, both porosity and the average pore diameter affected the mechanical properties of aerated concrete.

**Figure 9:**
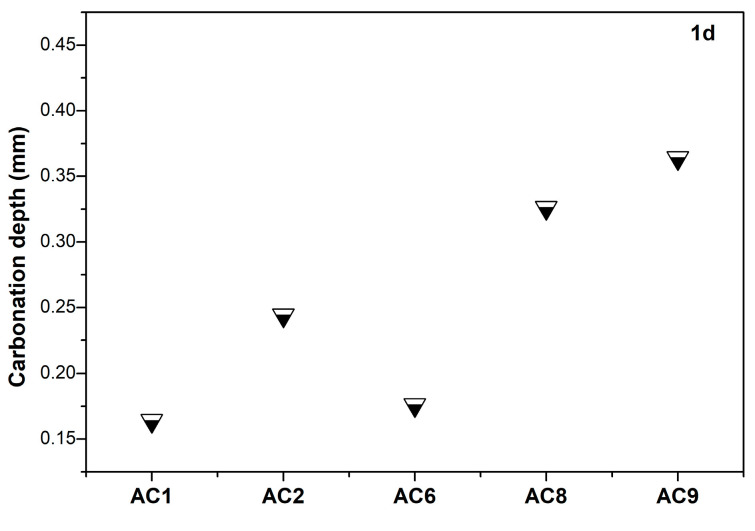
The carbonation depth of aerated concrete containing spodumene mining residue after steam curing for 48 h and carbonation curing for 1 d.

Pore size and distribution of cement-based materials significantly affected the transport characteristic of water and gas, which in turn affected its carbonization rate. The carbonization-resistance property of aerated concrete containing spodumene mining residue was studied. Figure 9 shows the carbonization depth of the aerated concrete after steam curing for 48h and carbonation curing for 1d. Compared to the other experimental groups, AC1 and AC6 had lower carbonation depth. The carbonation process of cement-based materials including carbon dioxide gas transferred to the pores of matrix, carbon dioxide gas was dissolved into the pore solution and formed carbonate. Carbonate was consumed in pore solution to form calcium carbonate, and calcium carbonate deposition [17,18,19]. Therefore, the pore structure, the pore saturation and pore solution composition of cement-based materials all affected the carbonation rate.

All samples were completely carbonized after carbonation curing for 3d. From another perspective, the aerated concrete containing spodumene mining residue might be more suitable for the carbonization curing method with the proper concentration of carbon dioxide.

With the development of the lithium battery industry, the amount of spodumene mining residue is increasing year by year. As an environmentally friendly material, the demand for aerated concrete is also increasing, under the background of carbon emission. The result of preparation and performance studies of aerated concrete with the SMR aggregate completely replacing quartz sand have confirmed the feasibility of SMR as siliceous materials, which reduced the aggregate cost of ordinary aerated concrete with quartz sand.

In addition, no significant leaching of harmful components from SMR have been found under an alkali environment. Then, the interaction mechanism between cement-based materials and SMR was further studied, in the following.

### 3.3. Interaction Between Cement Paste and Spodumene Mining Residue

Table 4 lists the mix proportion and the results of the activity-index experiment of spodumene mining residue powder. The groups SMR-30 and SMR-50 refer to 30 wt% and 50 wt% replaced by spodumene mining residue powder, respectively. Compared with the control group, the activity index of spodumene mining residue powder was lower than 70%. The higher the amount of spodumene mining residue powder replacing cement, the lower its activity index. It was proved that the spodumene mining residue powder might had weak secondary hydration activity in the alkaline medium provided by the cement matrix. There might be an interaction between spodumene mining residue powder and cement paste [20,21].

The activity index of spodumene mining residue was tested according to Chinese standard GB/T 1596-2017 [22]. In order to further investigate the interaction between spodumene-mining-residue powder and cement paste, the hydration reaction of spodumene-mining-residue powder was studied by soaking in simulated-pore solution. Table 5 lists the mix proportion of simulation-pore solution. Four groups of simulated-pore solutions K1 and K4 were prepared, according to Table 5. Spodumene mining residue was added into the simulated-pore solution, fully stirred, and left for a certain time. After 2 h of reaction, a kind of white flocculent began to appear in the beaker, attached to the bottom of the cup and the surface of the spodumene-mining-residue particle. Then, the white reaction product was obtained by centrifugation, cleaned with ultrapure water, and dried in a vacuum drying oven.

**Figure 10:**
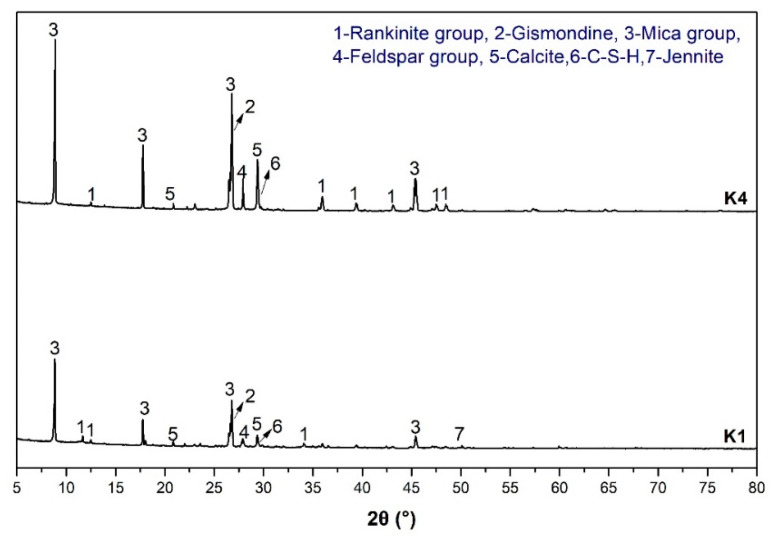
XRD pattern of the products produced by simulation-pore-solution alkali-leaching spodumene-mining residue.

The products in the simulation pore solution were analyzed by XRD, FTIR and TG. Compared with K4, simulation pore solution K1 had a higher pH and a certain amount of potassium and sodium ions. Figure 10 shows the XRD pattern of the products in two simulation pore solutions containing spodumene-mining-residue particles. The mineral composition containing the rankinite group, gismondine, mica group, feldspar group, calcite, C-S-H and jennite were observed in the XRD patterns of K1 and K4. Among them, the rankinite group included xonotlite and tobermorite phases, and the mica group included white mica and phlogopite. The different pH and ion concentrations of K1 and K4 resulted in a slight difference in mineral crystallization degree and type of products produced by alkali-leaching spodumene-mining residue. The jennite was only found in K1 simulated-pore solution. The characteristic peaks of gismondine, calcite and feldspar in K4 were higher than that in K1. This confirmed that the pH value and the concentration of other ions in pore solution significantly affected the crystallization degree of the crystalline phase [23].

**Figure 11:**
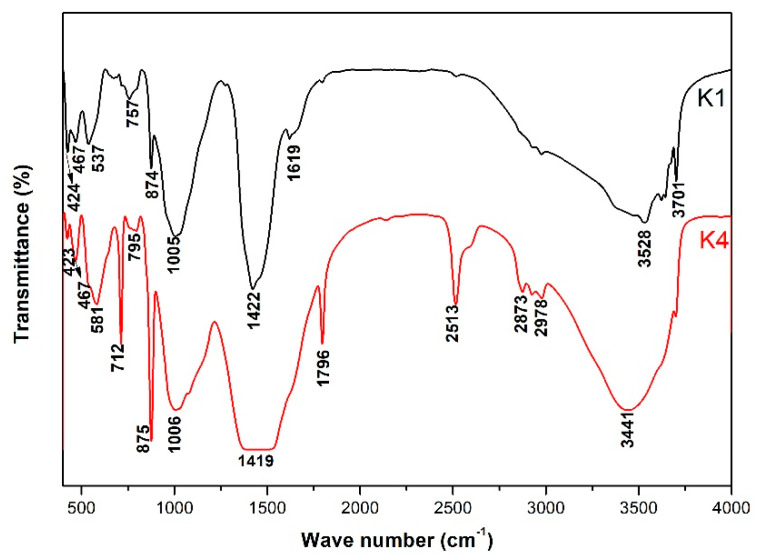
FTIR of the products produced by simulation-pore-solution alkali-leaching spodumene-mining residue.

Figure 11 shows the FTIR analysis result of the products of alkali-leaching spodumene-mining residue in simulation pore solution. FTIR spectra bands in the region 467 cm^−1^ corresponded to the bending vibration of the Si-O [24]. Bands in the region 350 cm^−1^ to 500 cm^−1^ and 700 cm^−1^ to 950 cm^−1^ were attributed to the characteristic peaks of [AlO_4_] and [AlO_6_] in the gismondine and mica group, respectively [25]. Bands around 713 cm^−1^, 875 cm^−1^, 1000 cm^−1^ and 1796 cm^−1^ corresponded to the vibration of CO_3_^2−^ in calcite, while bands at 1600 cm^−1^ to 1800 cm^−1^ corresponded to the characteristic peak of the carbonyl group [26]. Bands at 3200 cm^−1^ to 3600 cm^−1^ matched the bending vibration and asymmetric stretching vibration of -OH, respectively. The peak at 3700 cm^−1^ corresponded to the symmetric stretching vibration of -OH in silicate. The difference in pH value and ion concentration leads to the difference in [Al-O] _n_^m−^ coordination number and [Si-O] _n_^m−^ polymerization degree, which leads to a wave-number shift. With the increase of coordination number, the vibration frequency of [Al-O]_n_^m−^ shifted towards the lower wavenumber. The increase of polymerization increased the absorption frequency of [Si-O] _n_^m−^. This led to different positions of vibration peaks for the same ionic groups of the same crystallization phases in K1 and K4 solutions.

**Figure 12:**
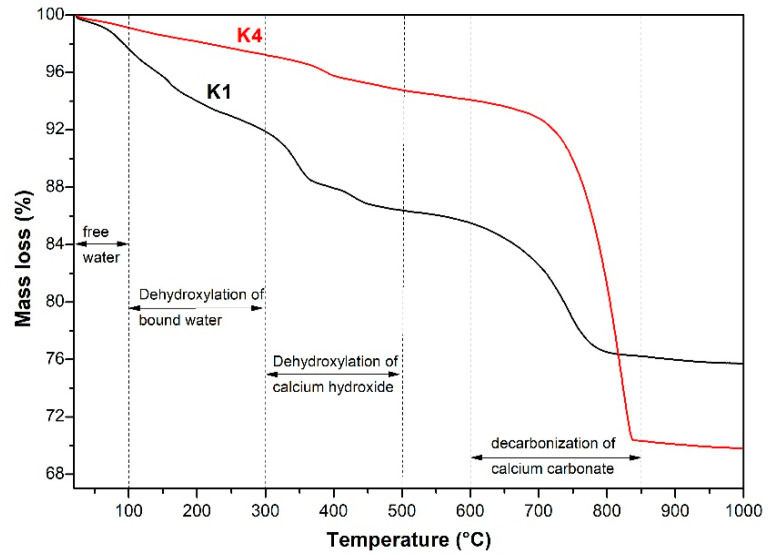
TG curve of the products produced by simulation-pore-solution alkali-leaching spodumene-mining residue.

Figure 12 shows the thermogravimetric analysis (TG) results of the products produced by simulation-pore-solution alkali-leaching spodumene-mining residue. With the increase in experimental temperature, four weightlessness zones were observed in the TG curve of alkaline leaching products. The initial weight loss was caused by the evaporation of free water. The second weightlessness zone between 100 °C and 300 °C was inferred to be the dehydration of bound water from C-S-H and C-(M)-S-H [27,28,29]. The third (300 °C to 500 °C) and the fourth (600 °C to 850 °C) weightlessness zones corresponded to the dihydroxy of CH and the decomposition of calcite [30,31], respectively.

Combined with the results of XRD, FTIR and TG analysis, the mineral phases of the products were calcium silicate hydrate (such as C-S-H and its derivatives, tobermolite), gismondine, mica and feldspar. Those phases were similar to the hydration products of cement, which proved that the spodumene mining residue still had weak pozzolanic activity, although it was used as the siliceous material in aerated concrete. The content of solid waste in aerated concrete exceeded 64 wt%, and the compressive strength of the 800 kg/m^3^-grade sample could still reach 2.5 MPa, which was due to the pozzolanic activity of spodumene mining residue.

Compared with K1, the K4-simulation pore solution had lower alkalinity and concentrations of potassium and sodium ions, which led to different polymerization of [Si-O] _n_^m−^ and coordination number of [Al-O]_n_^m^. This led to silico–calcium hydration products with similar chemical composition and different structures, such as C-S-H, tobermorite and jennite. The hydrated silicate monomer, which was continuously generated by cement hydration, polymerized with the protonated dimeric C-S-H, forming a small amount of long-chain C-S-H. This was the main reason for the increase in C-S-H polymerization degree with the increase of curing age. At the same time, in the presence of aluminum, it formed higher polymerized aluminum–silicon chains of bridging dimeric C-S(A)-H [32,33]. The formation of C-S-H in cementitious materials is critical to their gain in strength. As one of the key parameters, pH is reported to affect the nucleation and growth of C-S-H. pH affected the formation of C-S-H mainly through the protonation of silicates, which determined the surface charge of silicate tetrahedra and consequent chemical composition of C-S-H [34,35]. At high pH, the formation of CaOH^+^ as the first step before the formation of C-S-H was responsible for the high Ca/Si ratio under such conditions.

**Figure 13:**
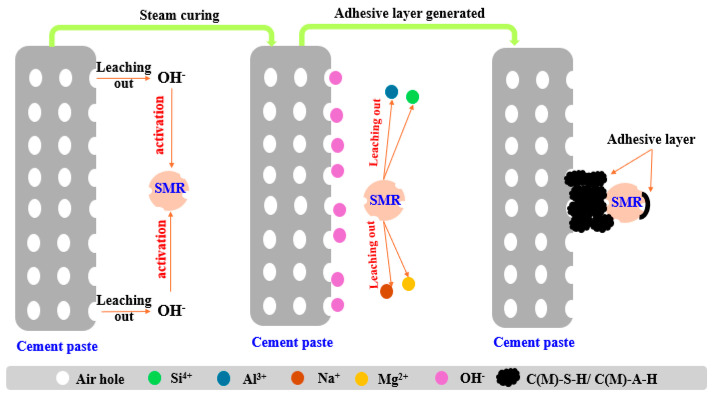
The mechanism diagram of interaction between spodumene mining residue and cement paste.

In sum, SMR particles had weak pozzolanic activity in an alkaline environment, which had the potential to improve the paste–aggregate interface structure. Figure 13 shows the chemical interaction between SMR particles and cement in an aerated concrete interface. The early hydration of cement provided an alkaline environment; then, Ca^2+^, Al^3+^ and Si^4+^ in the amorphous phase of SMR leached out and produced C-S-H or its derivatives, which was considered the secondary hydration of SMR. Part of the newly generated C-S-H adhered to the surface of SMR and filled the interface gap between SMR and cement paste, which was similar to the self-healing process of concrete.

## 4. Conclusions

This study evaluated the structure and properties of aerated concrete using spodumene mining residue as siliceous material. Spodumene mining residue improved the properties of aerated concrete through both physical and chemical processes.

(1) It was feasible to produce aerated concrete with superior mechanical properties and excellent pore structure using spodumene mining residue as siliceous material, completely replacing quartz sand.
(2) The interfacial compactness and mechanical properties of aerated concrete were improved by the secondary hydration of SMR.
(3) In an alkaline environment, the active ions Ca^2+^, Si^4+^ and Mg^2+^ in spodumene mining residue leached out and generated C-S-H (or its derivatives), which adhered to the surface of SMR and filled the gap between the spodumene mining residue particles and cement paste.

## Figures and Tables

**Table 1:** The chemical composition of raw materials.

Raw Materials	SiO_2_	Al_2_O_3_	CaO	Fe_2_O_3_	K_2_O	Na_2_O	TiO_2_	MgO	SO_3_	P_2_O_3_	Others
SMR	66.38	16.11	5.66	5.23	3.11	1.13	0.87	0.79	0.27	0.22	0.23
CE	18.17	4.34	63.15	3.09	0.8	0.11	0.3	0.87	4.68	0.25	4.24
LI	1.17	0.41	97.31	0.08	-	-	-	0.72	0.15	-	0.16
GY	3.57	0.71	41.83	0.53	0.30	0.09	0.09	0.42	52.12	0.02	0.32
SF	91.33	0.7	0.96	4.64	1.07	0.12	-	-	0.65	0.13	0.40

SMR, spodumene mining residue. CE, Portland cement. LI, lime. GY, gypsum. SF, silica fume.

**Table 2:** The purity of chemical reagents.

Chemical Drugs	NaOH	KOH	Ca(OH)_2_	Na_2_SO_4_	Na_2_SiO_3_
Purity/%	98	99.7	99.5	99	99

**Table 3:** Mix proportion of orthogonal experiments.

Code	CE/%	LI/%	SF/%	SMR/%	AP/%	W/S	GY/%	Curing Condition
AC1	20	12	0	68	0.08	0.50	2	Curing temperature: 20 °C, 40 °C, 80 °C Curing time 48 h
AC2	20	12	2	66	0.10	0.55	2	
AC3	20	12	4	64	0.12	0.60	2	
AC4	20	12	0	68	0.08	0.55	2	
AC5	20	12	2	66	0.10	0.60	2	
AC6	20	12	4	64	0.12	0.50	2	
AC7	20	12	0	68	0.08	0.60	2	
AC8	20	12	2	66	0.10	0.50	2	
AC9	20	12	4	64	0.12	0.55	2	

CE, cement. LI, quicklime. SF, silica fume. AP, aluminum powder paste. GY, gypsum. W/S, the ratio of water to solid. SMR, spodumene mining residue.

**Table 4:** The mix proportion and result of activity-index experiment of spodumene-mining-residue powder.

Code	Cement/g	Spodumene-Mining-Residue Powder/g	Standard Sand/g	Water/g	Activity Index (%)	
					7 d/g	28 d/g
Control group	450	0	1350	225	100.00	100.00
SMR-30	315	135	1350	225	68.35	56.33
SMR-50	225	225	1350	225	42.40	38.26

**Table 5:** The mix proportion of simulation pore solution.

Code	Concentration/mol·L^−1^					pH	Reaction Temperature/°C
	Ca(OH)_2_	NaOH	KOH	Na_2_SO_4_	Na_2_SiO_3_		
K1	0.001	0.2	0.6	0	0	13.63	80
K4	Saturation	0	0	0	0	12.54	80

## Data Availability

The original contributions presented in the study are included in the article, further inquiries can be directed to the corresponding author.
